# A Primary Spinal Cord Mixed Culture Method for In Vitro Analysis of Glial Heterogeneity and Inflammatory Responses

**DOI:** 10.1007/s11064-026-04777-9

**Published:** 2026-05-21

**Authors:** Cinthia Cristina de Oliveira Santos Costa, Catarina de Jesus Nunes, Ana Clara Neves Reis Pedreira, Silvia Lima Costa, Ravena Pereira do Nascimento

**Affiliations:** Laboratory of Neurochemistry and Cellular Biology, Institute of Health Sciences, Federal University of Bahia, Av. Reitor Miguel Calmon S/N, Salvador, 40231-300 BA Brazil

**Keywords:** Primary culture, In vitro models, Spinal cord, Neuroinflammation, Astrocyte heterogeneity

## Abstract

Traditional in vitro models used in spinal cord research often rely on simplified cell systems, such as PC-12 cells, which do not reproduce the mixed cellular environment or glia-neuron interactions of spinal cord tissue. Although primary spinal cord cultures have been described, many protocols focus on purified populations and require technically demanding or costly procedures. In this study, we established a low-cost mixed primary spinal cord culture from neonatal Wistar rats and characterized its cellular composition and inflammatory responsiveness in vitro. At 7 days in vitro, immunofluorescence analysis showed the presence of neurons (42.5%), astrocytes (17.2%), and microglia (4.4%), together with an unresolved non-labeled cell population (35.9%). GFAP-positive astrocytes displayed marked morphological heterogeneity, including process-bearing, flattened, and ramified forms. To examine the inflammatory responsiveness of the preparation, cultures were exposed to lipopolysaccharide (LPS, 5 µg/mL for 12 h), which increased nitrite levels and was associated with a higher abundance of amoeboid-like cells and increased Iba1-positive and CD68-positive cell labeling. The culture could also be maintained for prolonged periods in vitro, although its biological interpretation changed over time as non-neuronal cells became more prominent. These findings support the use of this preparation as an accessible mixed primary spinal cord platform for studying glial-associated inflammatory responses in vitro and for future screening of anti-inflammatory compounds.

## Introduction

Spinal cord injury (SCI) is a condition with high morbidity and mortality, mainly affecting adults, with profound impacts on global health and the economy. One of the major challenges in the treatment of SCI is to promote tissue regeneration in damaged nerve tissue [18, 51]. The primary mechanical insult is invariably followed by a complex secondary injury cascade, in which neuroinflammation, oxidative stress, and excitotoxicity exacerbate the initial damage and contribute to permanent functional deficits.

Two glial cell types stand out in the pathophysiology of SCI: astrocytes and microglia. These cells are critically involved in both the early inflammatory response and later tissue remodeling. Astrocytes are predominant glial cells that tile the central nervous system (CNS) [6, 17]. According to Baldwin et al. [6], the complex morphology of astrocytes has evolved to allow them to contact and signal with diverse cells at different distances to sample, regulate, and contribute to the extracellular environment and participate widely in cell-cell signaling during physiology and disease. After SCI, they become reactive and undergo morphological and functional changes that contribute to injury repair or further exacerbate neurodegenerative processes, depending on factors such as the cytokines and chemokines they release into the inflammatory microenvironment, the recruitment of immune cells to the site of injury, and the formation of the glial scar [11, 38, 39].

Microglia are the resident immune cells of the CNS, acting as sentinels that are activated in response to injury or foreign agents. Like astrocytes, microglia play a dual role: they can clear debris and secrete neuroprotective factors but may also contribute to chronic inflammation and thereby amplify secondary injury if their activation becomes dysregulated [14, 40]. In the context of SCI, the neurochemical landscape is further complicated by the release of inflammatory mediators such as nitric oxide (NO). The activation of the CD14/Toll-like receptor 4 (TLR4) pathway triggers an intracellular signaling cascade that culminates in the upregulation of pro-inflammatory mediators, creating a neurotoxic environment for spinal neurons [9].

The utilization of primary spinal cord cultures in research is relatively uncommon, in part because of the technical difficulty of spinal cord dissection and the relatively low cellular yield [21, 28], with most studies focusing primarily on the isolation of motor neurons via expensive and technically demanding protocols [30, 35]. Extant literature frequently employs PC-12 cells as an in vitro model to study SCI [49, 52]. However, given the origin of these cells, they do not represent the specialized neurochemical characteristics, architectural complexity, or the glia-neuron crosstalk present in the spinal cord [48]. Standard models of inflammatory damage often utilize hydrogen peroxide, LPS, or kainic acid to induce these pathological states in simplified cell lines [22, 44, 46, 50].

In this context, accessible primary in vitro models may provide a useful intermediate system between simplified monocultures and in vivo [15] injury models. Such preparations offer a controlled environment for investigating cellular and molecular mechanisms, support early screening of potential neuroprotective agents, and may contribute to the Reduction, Replacement, and Refinement of animal experimentation when used appropriately [43, 47]. However, their biological scope must be defined carefully, since in vitro systems do not reproduce the full complexity of SCI in vivo.

In this study, we established a low-cost primary spinal cord culture from neonatal rats that preserves a mixed cellular environment for experimental analysis at 7 days in vitro and can be maintained for prolonged periods under culture conditions. Using lipopolysaccharide as a controlled trigger of TLR4-mediated inflammatory signaling, rather than as a full surrogate of SCI [9], we characterized the cellular composition and morphological heterogeneity of this culture at the acute experimental time point. We aimed to evaluate this preparation as a practical primary spinal cord platform for studying glial-associated neuroinflammatory responses in the presence of neuronal and non-neuronal cell populations, and as a potentially useful system for future neuroprotective screening.

## Materials and Methods

### Primary Culture of Spinal Cord Cells

The procedures were conducted in accordance with the standards set forth by the Commission on Ethics in the Use of Animals (CEUA) of the Institute of Health Sciences of the Federal University of Bahia (process number 7326091122). All experimental conditions were analyzed using at least three technical replicates and were repeated in at least three independent culture preparations, when indicated.

The spinal cord extraction was performed based on the method described by Malon and Cao [32] with modifications. Primary spinal cord cultures were prepared from 1 to 2-day-old Wistar rats. The animals were euthanized by decapitation, and a longitudinal incision was made to expose and remove the spinal column. The spine was placed in a 3 × 10 cm diameter Petri dish containing a cold balanced Hank’s salts solution (HBSS). The spinal cord was then isolated and minced into fine pieces. Next, the tissue was incubated in trypsin solution for enzymatic dissociation. The tissue was maintained in a humidified atmosphere with 5% CO₂ at 37 °C for 10 min. After digestion, the suspension was centrifuged for 5 min at 2000 rpm, and the supernatant was discarded. Subsequently, the cells were resuspended in DMEM F12 medium (Gibco,12400-016), which had been supplemented with 10% bovine fetal serum (Gibco, 12657), 10% equine serum (Gibco, 16050122), penicillin (100 U/mL) and streptomycin (100 µg/mL) (both from Cultilab, Brazil), as well as 0.1% B27 (Gibco, 17504044). After that, the cells were distributed in culture plates previously sensitized with poly-D-lysine (10 µg/mL) and laminin (2.5 µg/mL) at a density of 3.0 × 10^4^ cells in 96-well plates (0.33 cm²) and 3.0 × 10^5^ in 24-well plates (2.0 cm^2^), contingent on the experiment. The culture medium was replaced every 48 h. For characterization of the primary culture and for assays of the lipopolysaccharide (LPS)-induced inflammatory response, cells were maintained for 7 days in vitro (DIV7). In parallel, satellite cultures were maintained for longer periods, up to 90 days in vitro (DIV90), to assess long-term persistence and gross morphological changes in culture. These long-term observations were distinct from the acute experimental assays performed at DIV7. For the control conditions, all experiments used DMEM-F12 culture medium supplemented as previously described.

### Determination of the LPS Concentration for Induction of Inflammatory Response

To induce an inflammatory response, primary spinal cord cultures were treated with Escherichia coli lipopolysaccharide (LPS, Sigma-Aldrich, USA) at DIV7. A stock solution of LPS was prepared in phosphate-buffered saline (PBS, pH 7.4) at a concentration of 1 mg/mL and stored at − 20 °C until use. Cultures were exposed to LPS at final concentrations of 0.1, 1, 2, 5, or 10 µg/mL for 12 h to determine the concentration to be used in subsequent experiments.

### Measurement of Nitric Oxide Release

The production of nitric oxide (NO) was quantified indirectly by measuring the concentration of nitrite accumulated in the culture medium using the Griess reaction. At the end of the treatment period, culture supernatants were collected and incubated with an equal volume of Griess reagent containing 1% sulfanilamide and 0.1% N-1-naphthylethylenediamine dihydrochloride (NED, G7921, Invitrogen) at room temperature. After 15 min, the absorbance of the samples was measured using a Varioskan Flash spectrophotometer (Thermo, Waltham, MA, USA). Nitrite concentrations were determined by comparison with a standard curve generated using sodium nitrite (NaNO₂) diluted in culture medium.

### Measurement of TNF-Alpha by ELISA

Tumor necrosis factor (TNF)-alpha levels were quantified in culture supernatants using a commercially available enzyme-linked immunosorbent assay (ELISA) kit (Invitrogen, KRC3011) according to the manufacturer’s instructions. Supernatants were collected after 12 h of treatment and added to the ELISA plate. After completing the assay, the absorbance was measured at the recommended wavelength using a microplate reader. TNF-α concentrations were calculated from a standard curve generated using the standards provided with the kit. All experimental conditions were evaluated in at least three independent culture preparations, and replicate wells from each preparation were averaged before statistical analysis.

### Characterization of Major Cell Populations in Primary Spinal Cord Culture by Phase-Contrast Microscopy and Immunostaining

Phase-contrast microscopy was used to monitor and morphologically characterize the primary spinal cord culture. Cells were monitored from day 0 (DIV 0) to 90 days in vitro (DIV90) using phase-contrast microscopy (Nikon microscope coupled to an imaging camera ).

Immunostaining was performed to characterize the major cell populations present in the culture. Classical markers for astrocytes (GFAP 1:500, Dako, Z0334; GFAP 1:800, Cell Signaling, 3670 S), microglia (Iba1, 1:200, Wako, 019-19741 and CD68, 1:100, Abcam, ab53444) and neurons (β-III-tubulin, 1:500, Biolegend, 801202 and anti-Chat, 1:100, Sigma, AMAB91130) were used. Briefly, after 7 days in vitro, cultures were fixed in ice-cold methanol for 20 min. The fixative was removed, and the cells were rehydrated in ice-cold phosphate-buffered saline (PBS) for 30 min. Cultures were then incubated with primary antibodies diluted in 1% bovine serum albumin (BSA) in PBS for 12 h at 4 °C in a humidified chamber.

After incubation with primary antibodies, the cultures were washed three times with PBS and incubated with the appropriate secondary antibodies diluted in 1% BSA in PBS for 2 h at room temperature. The secondary antibodies used were Alexa Fluor 488 goat anti-mouse IgG (1:1000, Invitrogen, A11001), Alexa Fluor 488 goat anti-rabbit IgG (1:1000, Invitrogen, A11008), Alexa Fluor 594 goat anti-mouse IgG (1:1000, Invitrogen, A11005), and Alexa Fluor 594 goat anti-rabbit IgG (1:1000, Invitrogen, A11012).

As negative controls, primary antibodies were omitted from the staining procedure. The cultures were then stained with the fluorescent agent *DNA* intercalator 4’,6’-diamino-2-phenylindole (DAPI, Eugene) at a concentration of 5 µg/mL for 10 min at room temperature. The cells were then analysed and photographed under a fluorescence microscope (Leica DMIL Led Fluor/Leica DFC7000 T camera). Eight micrographs were taken for each condition. Quantification was performed using ImageJ 1.33u (National Institute of Health, USA). In addition, cell morphology was analyzed by skeleton reconstruction using ImageJ-Win64 and the Analyze Skeleton (2D/3D) plugin, as previously described [5].

### MTT Assay

Cell viability was assessed using the 3-(4,5-dimethylthiazol-2-yl)-2,5-diphenyltetrazolium bromide assay, MTT (Sigma, St. Louis, MO, USA), based on the reduction of yellow tetrazolium salt to purple formazan by metabolically active cells, as previously described by Hansen et al. (1989). Spinal cord cells were seeded at a density of 3.0 × 10^4^ cells per well in 96-well plates. At 7 days in vitro, cultures were treated with LPS at concentrations of 0.1, 1, 2, 5, or 10 µg/mL for 12 h. Control cultures used DMEM-F12 medium supplemented as previously described. After treatment, cells were incubated with MTT solution at a final concentration of 1 mg/mL for 2 h. The cells were then lysed, and the formazan crystals were solubilized in a solution containing 20% weight per volume sodium dodecyl sulfate and 50% volume per volume dimethylformamide at pH 4.7 during overnight incubation. Optical density was measured at 595 nm using a Varioskan Thermo Plate Reader (Thermo Fisher Scientific, Vantaa, Finland). All experimental conditions were evaluated in at least three independent replicates.

### Quantitative Analysis and Cell Counting

To characterize the cell population and inflammatory response, quantitative data were obtained using a standardized sampling protocol. For each experimental condition, 15 distinct microscopic fields were analyzed, sampled from five images per coverslip across three independent experiments. Cell counting was performed using Fiji, the ImageJ distribution [45]. The percentages of specific cell populations, including microglia, astrocytes, and neurons, were calculated relative to the total number of cells, determined by DAPI-positive nuclei, to account for the spatial heterogeneity inherent to primary spinal cord cultures.

### Statistical Analysis

Statistical analyses were performed using GraphPad Prism software version 5.00 for Windows. Data were initially evaluated for Gaussian distribution. The Kruskal-Wallis test, followed by Dunn’s post hoc test, was employed to analyze non-parametric data. Parametric data were analyzed using one-way analysis of variance (ANOVA), followed by Tukey’s post hoc test. A 95% confidence level was adopted, and *p* < 0.05 was considered statistically significant. The statistical test used for each analysis is indicated in the corresponding Results section or figure legend. For each independent culture preparation, measurements obtained from replicate wells were averaged before statistical analysis, and the resulting mean value from each preparation was used as the experimental unit.

## Results

### Primary Spinal Cord Cultures can be Maintained In Vitro for up to 90 Days

**Fig. 1:**
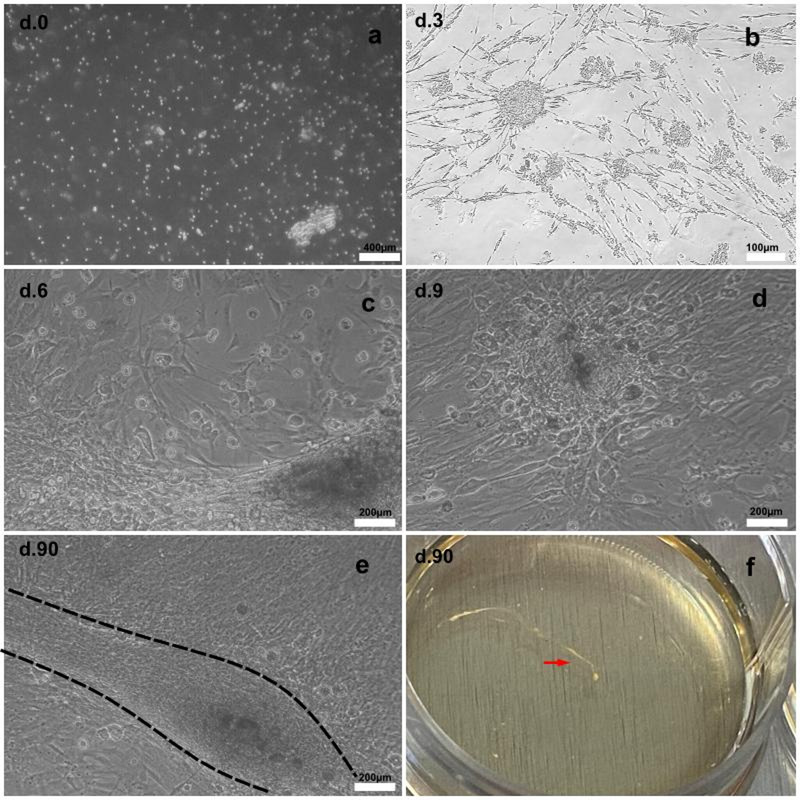
Time course of primary spinal cord development. Phase-contrast photomicrographs showing the development and maturation of the primary culture. **a** Adherent cells observed at DIV0. **b** Formation of cell clusters at DIV3. **c** Expansion of primary spinal cord cells from these clusters at DIV7. **d** Higher-magnification view of the cell clusters presents in culture. **e** Mature satellite culture at DIV90 showing dense fiber-like structures, highlighted by dotted lines. **f** Macroscopic view of dense fiber-like structures visible at DIV90, indicated by red arrows

As early as 3 h after plating, cells were observed adhered to the bottom of the culture plate. Residual tissue fragments appeared to provide physical support for cell attachment and outgrowth, and the culture expanded as discrete cellular clusters that progressively increased in size over time. Under these conditions, satellite cultures could be maintained for up to 90 days in vitro and retained gross structural integrity by phase-contrast observation (Fig. 1).

### Primary Spinal Cord Cultures Contain Multiple Identifiable Cell Populations and Cell Growth Aggregates

**Fig. 2:**
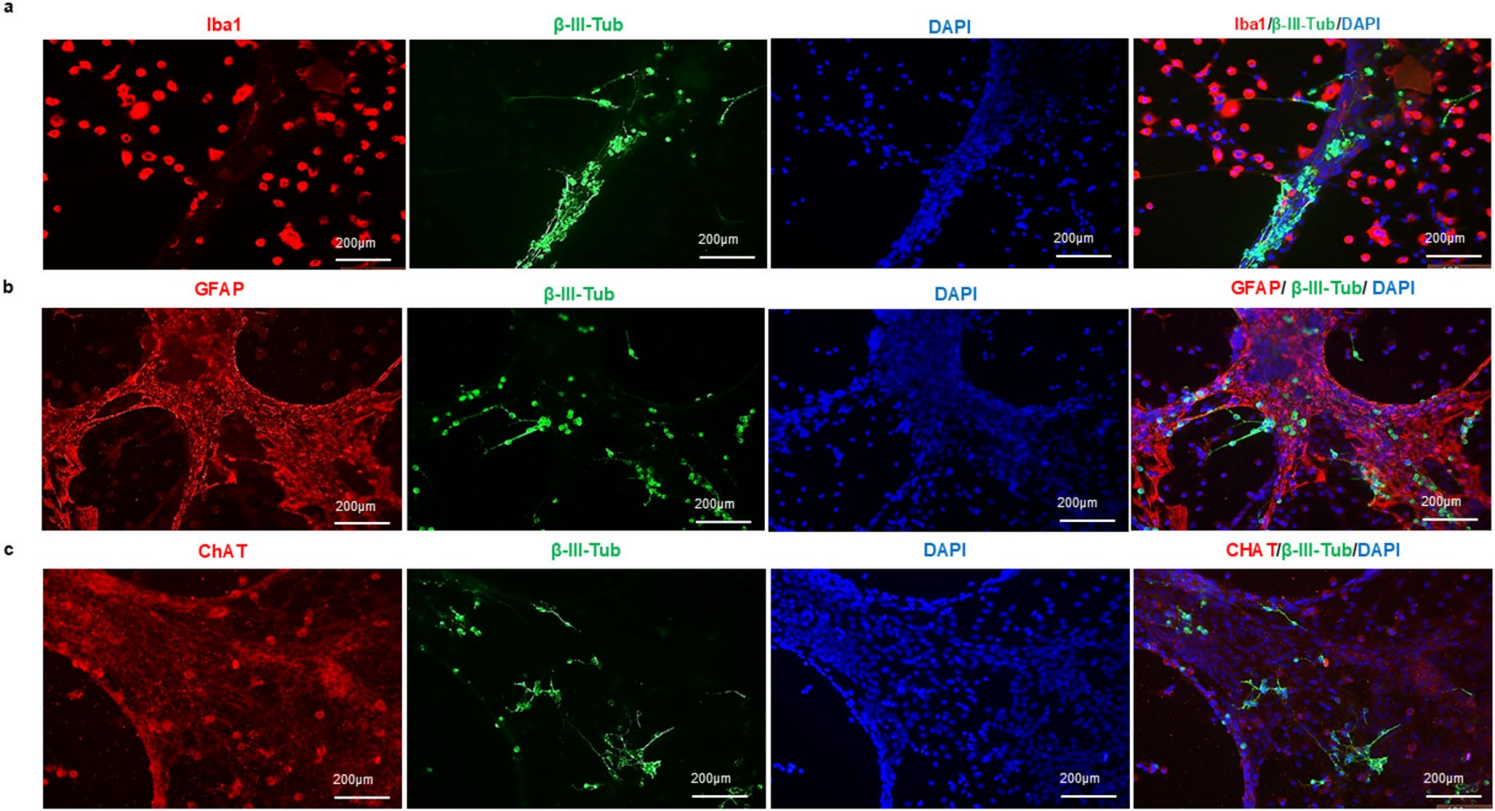
Immunofluorescence characterization of the primary spinal cord culture at DIV7. Representative images showing cells labeled with Iba1 and βIII-tubulin (**a**), GFAP and βIII-tubulin (**b**), and ChAT and βIII-tubulin (**c**). Cell nuclei were counterstained with DAPI

At 7 days in vitro, immunofluorescence analysis confirmed the presence of neurons, astrocytes, and microglia in the primary spinal cord culture (Fig. 2). Quantification relative to the total number of DAPI-positive nuclei showed that cells positive for neuronal markers, defined by βIII-tubulin and ChAT immunoreactivity, represented 42.50% of the total cell population. GFAP-positive astrocytes accounted for 17.20%, Iba1-positive microglia accounted for 4.40%, and the remaining 35.90% corresponded to cells not labeled by the markers used in this study and were therefore classified conservatively as unresolved cells (Table 1).

**Fig. 3:**
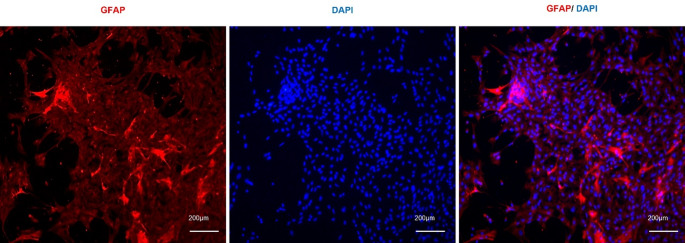
GFAP-positive astrocytic organization in primary spinal cord culture at DIV7. Representative image showing GFAP-positive cells distributed throughout the culture in a fibrillar pattern. Cell nuclei were counterstained with DAPI

Phase-contrast observation indicated that neuronal profiles were more evident during the first week in culture, whereas non-neuronal cells became progressively more prominent at later time points. At DIV7, GFAP-positive cells displayed an organized distribution within the culture and formed an extended fibrillar network throughout the preparation, often surrounding or interposed among other cells (Fig. 3).

**Table 1:** The relative number of neurons and glial cells in primary spinal cord culture at 7 days in culture

Cell type	Amount (%)
Neuron (Class III β-tubulin-positive neurons)	7.50
Neuron (ChAT-positive neurons)	35
Astrocyte (GFAPChAT-positive cells)	17.20
Microglia (Iba1-positive cells)	4.40
Other cells (e.g., fibroblast-type cells, endothelial cells)	35.90
Total	100

**Fig. 4:**
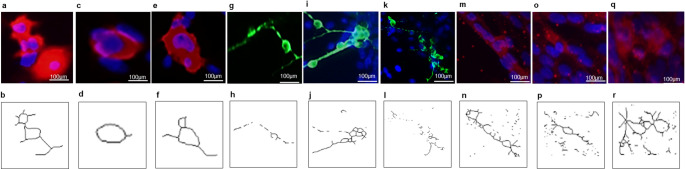
Morphological features of microglia and neurons in primary spinal cord culture. Representative immunofluorescence images and corresponding skeleton reconstructions generated with ImageJ and the Analyze Skeleton 2D and 3D plugin. Iba1-positive cells predominantly displayed amoeboid-like morphology (**a**–**f**). βIII-tubulin-positive neurons showed rounded somata with fine processes (**g**–**l**). ChAT-positive neurons displayed comparatively larger somata with extending processes (**m**–**r**)

**Fig. 5:**
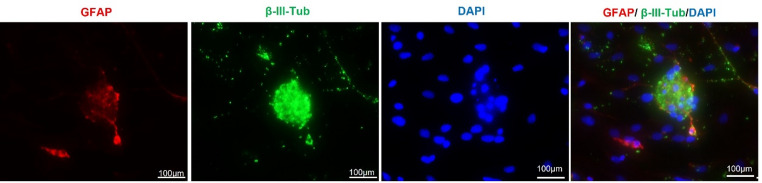
Cell growth aggregates in primary spinal cord culture. Representative image showing cell aggregates containing GFAP-positive astrocytes and βIII-tubulin-positive neurons. Cell nuclei were counterstained with DAPI

βIII-tubulin-positive neurons typically displayed a rounded soma with fine processes. In contrast, ChAT-positive neurons exhibited larger somata with processes extending from the cell body (Fig. 4). In addition to isolated neurons and glial cells, the primary spinal cord culture also contained cell growth aggregates that were evident from the first days in vitro. These aggregates contained cells positive for neuronal and astrocytic markers, indicating that they were composed, at least in part, of mixed cellular elements within the culture (Fig. 5). Some cells also displayed directed growth patterns along a single plane or across multiple planes, although these features were not quantified systematically.

### Astrocytes in Primary Spinal Cord Culture Display Morphological Heterogeneity

**Fig. 6:**
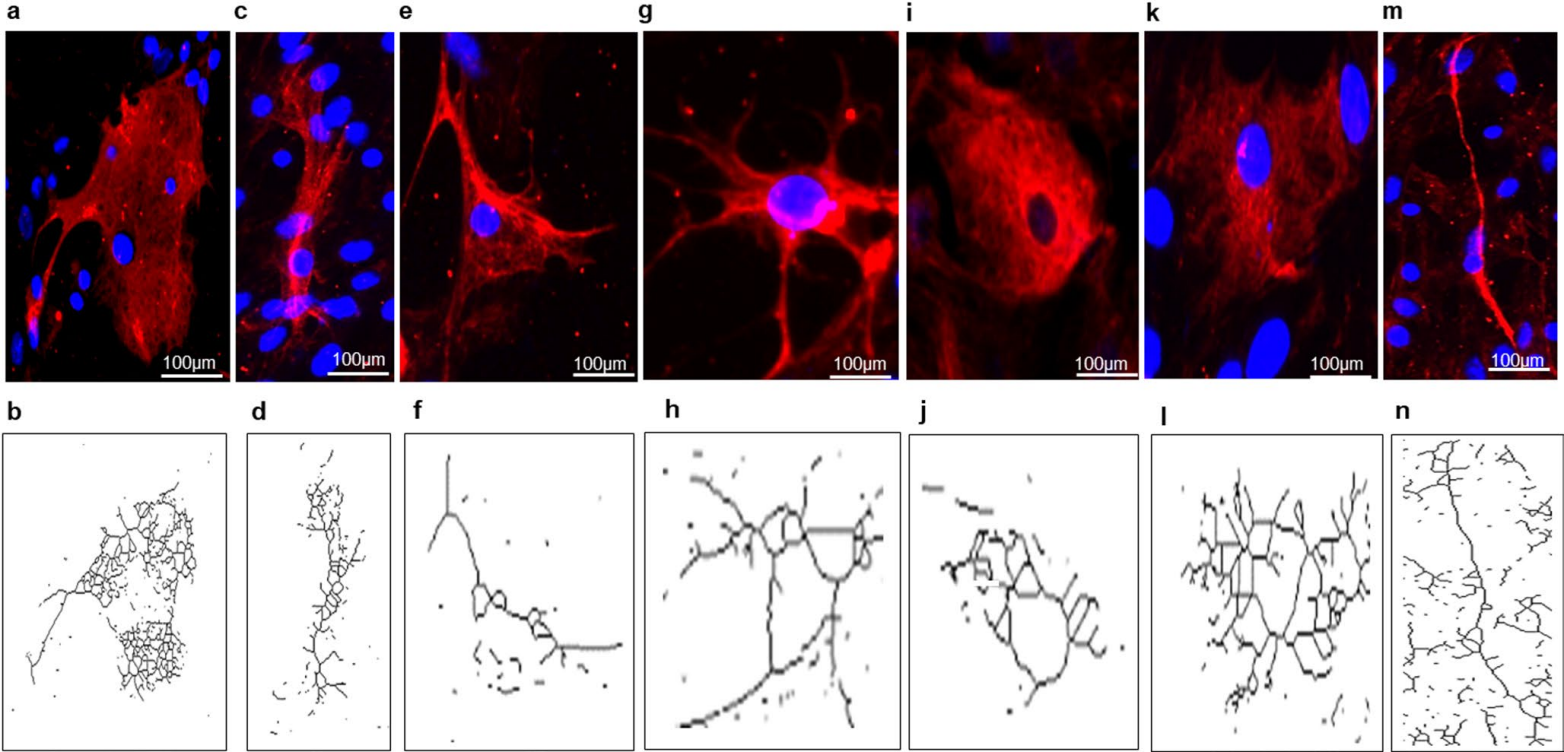
Representative GFAP-positive astrocytic morphologies in primary spinal cord culture. Immunofluorescence images and corresponding skeleton reconstructions generated with ImageJ and the Analyze Skeleton 2D and 3D plugin. The culture showed a range of astrocytic forms, including flat fibroblast-like cells (**a**, **b**), elongated cell bodies with short processes (**c**, **d**), triangular cell bodies with three short processes (**e**, **f**), cells with multiple thin elongated processes (**g**, **h**), rounded cells (**i**, **j**), cells with short thick processes (**k**, **l**), and cells with slender somata and long thin processes (**m**, **n**)

To characterize astrocytic heterogeneity within the culture, cells were labeled with GFAP. After one week in vitro (DIV7), the astrocytic population showed marked morphological diversity. Across cultures, we observed multiple astrocytic forms, including polygonal, flattened pancake-like, and more highly ramified cells, consistent with the range of morphologies previously described in neonatal spinal cord cultures [10, 26, 33]. Representative examples of these morphologies and their corresponding skeleton reconstructions are shown in Fig. 6a–n. These observations indicate that the culture preserves astrocytic morphological heterogeneity in vitro. However, these forms are interpreted here as evidence of phenotypic diversity and plasticity within the preparation rather than as proof of distinct functional specialization.

### Effect of LPS on Primary Spinal Cord Culture

**Fig. 7:**
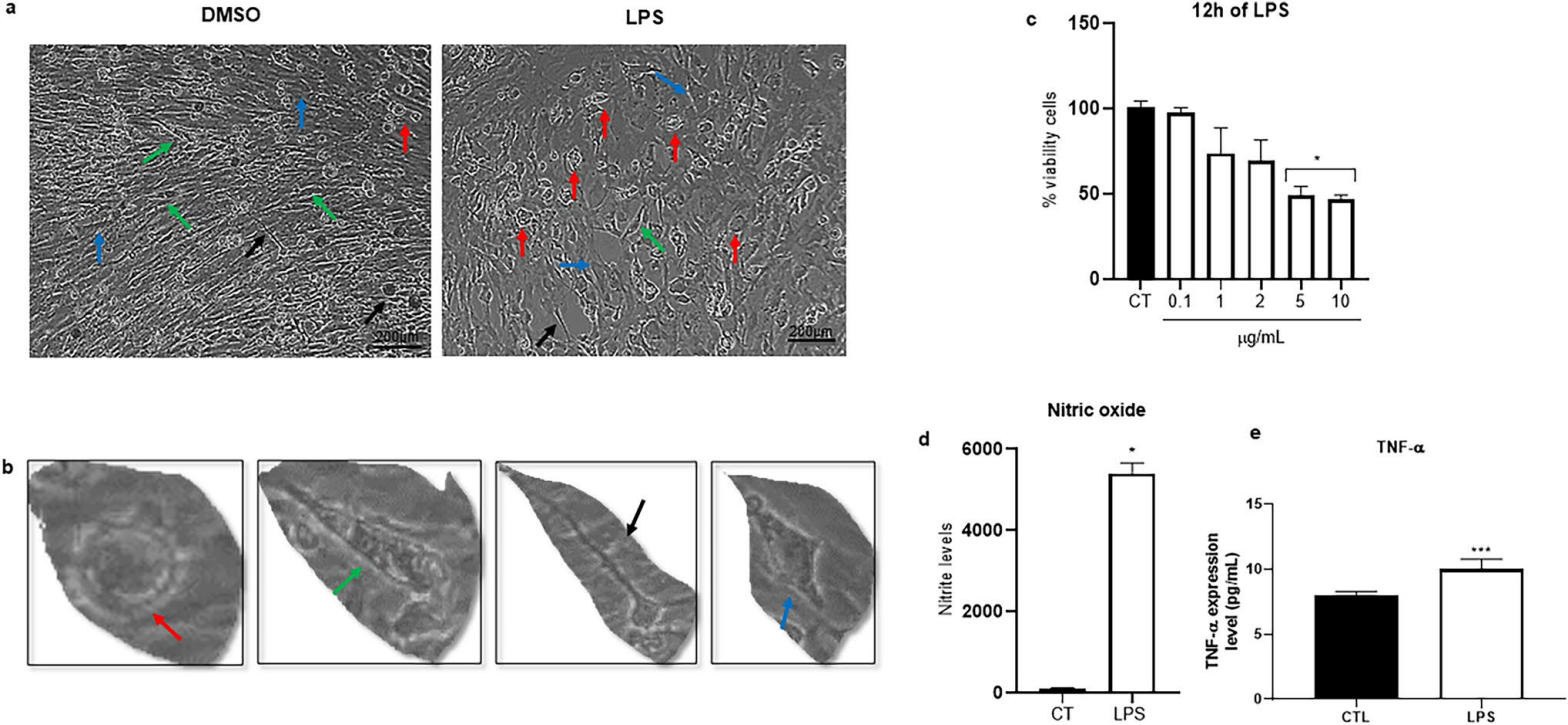
Effects of LPS on primary spinal cord culture. Phase-contrast photomicrographs showing representative morphological features of the culture after exposure to LPS at 5 µg/mL for 12 h (**a**, **b**). In panel **b**, red arrows indicate amoeboid-like cells, green arrows indicate fibroblast-like cells, black arrows indicate cells with thin processes, and blue arrows indicate polygonal cells. Panel **c** shows the effect of LPS at concentrations from 0.1 to 10 µg/mL for 12 h on overall culture metabolic activity, assessed by the MTT assay. Panel **d** shows nitrite levels in the culture supernatants after LPS treatment as an indirect measure of nitric oxide production. Panel **e** shows TNF-α levels in culture supernatants after vehicle or LPS treatment, as determined by ELISA. Data are presented as mean ± SEM from three independent culture preparations. For each preparation, replicate wells were averaged before statistical analysis. Panel **c** was analyzed using the Kruskal-Wallis test followed by Dunn’s post hoc test. Panel **d** was analyzed using the Mann-Whitney test. *p* < 0.05 versus control. Panel **e** t-test, *p* = 0.0006

Exposure to LPS for 12 h produced concentration-dependent effects in the primary spinal cord culture. The concentrations of 5 and 10 µg/mL were associated with a significant reduction in overall metabolic activity, as assessed by the MTT assay (Fig. 7c). Based on this result, 5 µg/mL LPS for 12 h was selected for subsequent inflammatory stimulation experiments.

Phase-contrast images showed morphological changes in LPS-treated cultures compared with control, including an increased number of amoeboid-like cells and disruption of the organized pattern of cell bundle growth (Fig. 7a, b). Nitric oxide production, estimated by nitrite accumulation in the culture supernatant, was significantly increased in the LPS-treated group compared with the control (Fig. 7d). TNF alpha levels were also significantly elevated in LPS-treated cultures compared with the control (Fig. 7e), further supporting the inflammatory responsiveness under these conditions.

**Fig. 7.1:**
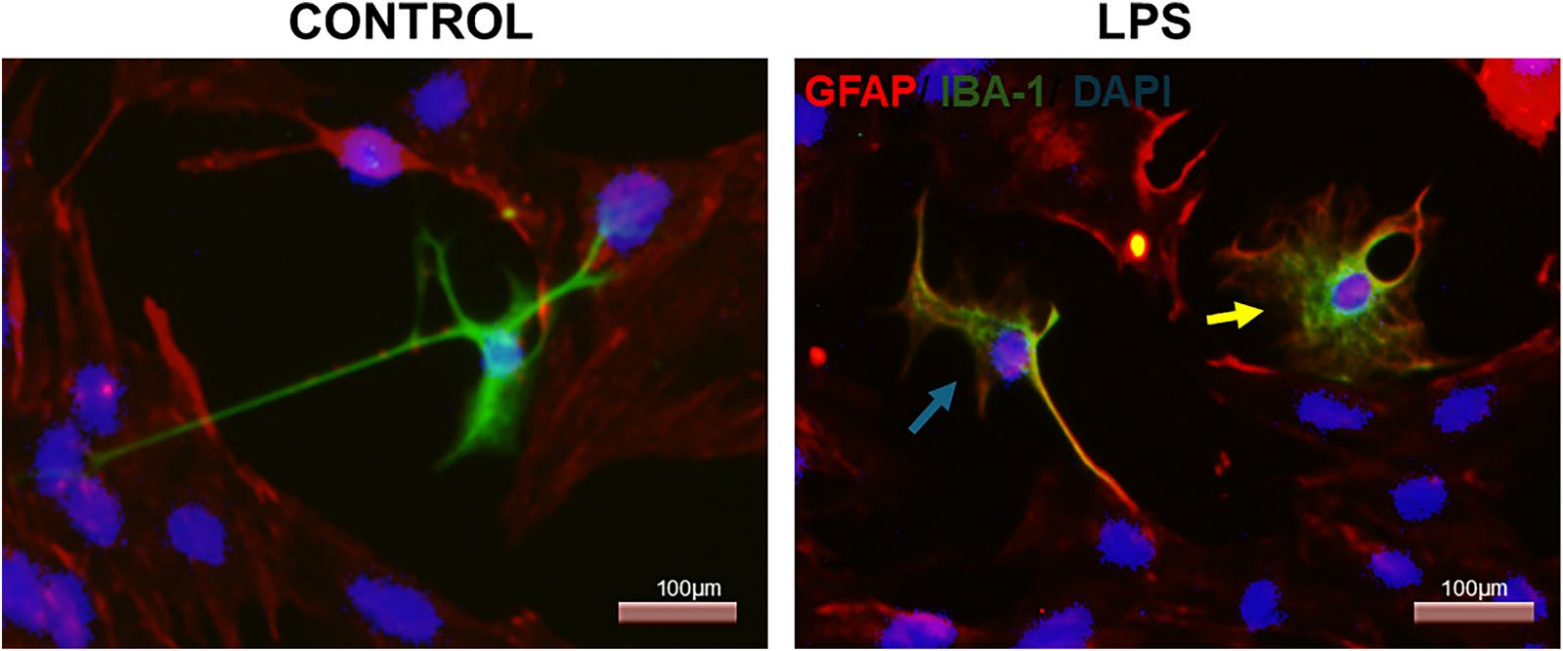
Effects of LPS on primary spinal cord culture. GFAP (astrocytes) and IBA-1 (microglia) labeling in primary spinal cord culture in a control condition and after LPS exposure. The fields are shown to illustrate the interaction between the two cell populations, as well as the morphological appearance of the labeled cells in each condition. In the control condition, microglial extensions establish contact with astrocytes, while in the LPS-treated condition, the blue arrow shows microglia in a transient state between branched and amoeboid, and the yellow arrow shows microglia with a highly rounded morphology, suggesting phagocytic and migratory capacity

The interaction of microglia with the astrocytic population is intimately observed, as seen in Fig. 7.1. At least two distinct morphologies of microglia can be observed, suggesting that these cells actively alter the shape of their processes and soma in response to different stimuli while maintaining direct contact with astrocytes.

### LPS Increases the Abundance of Microglia-Like and CD68-Positive Cells in Primary Spinal Cord Culture

**Fig. 8:**
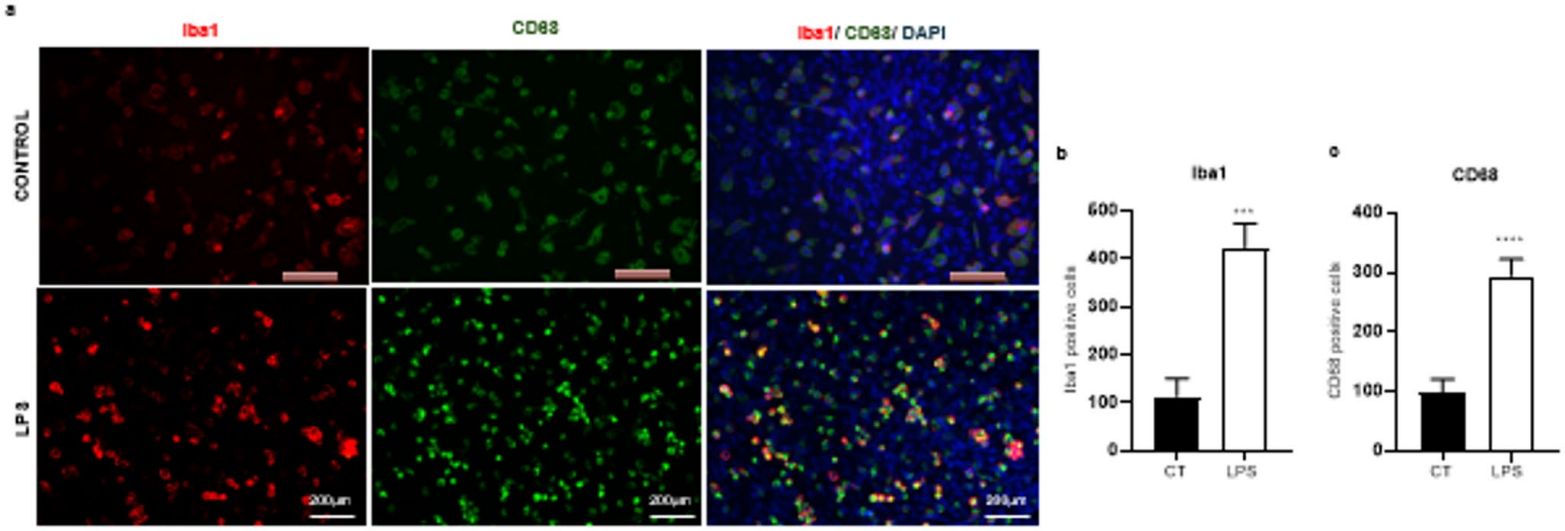
Iba1 and CD68 labeling in primary spinal cord culture after LPS exposure. **a** Representative immunofluorescence images showing Iba1 and CD68 labeling in control conditions and LPS-treated cultures. Representative fields are shown to illustrate the morphological appearance of the labeled cells under each condition. **b** Quantification of the percentage of Iba1-positive cells relative to the total number of DAPI-positive nuclei. **c** Quantification of the percentage of CD68-positive cells relative to the total number of DAPI-positive nuclei. Data are presented as mean ± SEM from three independent culture preparations. For each preparation, replicate wells were averaged before statistical analysis. Panel b was analyzed using the Mann-Whitney test, and panel c was analyzed using Student’s t test. ***p < 0.0006 and ****p < 0.0001 versus vehicle control. Scale bar = 200 μm

After treatment with LPS at 5 µg/mL for 12 h, phase-contrast observation showed an increased number of amoeboid-like cells compared with the control (DMEM - F12). Immunofluorescence analysis further showed an increased number of Iba1-positive cells and CD68-positive cells in LPS-treated cultures relative to controls (Fig. 8). Because CD68 was used here as a marker of phagocytic or lysosomal activation rather than as a lineage marker alone, these findings were interpreted as consistent with an enhanced inflammatory and phagocytic-like profile in the culture after LPS exposure.

## Discussion

Establishing a biologically relevant in vitro platform is important for investigating the neurochemical and neuroinflammatory mechanisms underlying spinal cord injury (SCI). In the present study, we developed a primary spinal cord culture that, unlike simplified monocultures or PC-12 cells, preserves a mixed cellular environment containing neurons and glial cells. Although previous studies have described the isolation of specific populations, such as motor neurons or purified astrocytes [1, 12, 30, 35], the present model retains these populations within the same preparation, allowing cell populations to coexist in a shared in vitro environment. At the same time, this culture should be interpreted as a simplified representation of the spinal cord microenvironment rather than as a full recapitulation of the secondary injury cascade, which in vivo also involves vascular disruption, infiltrating immune cells, edema, excitotoxicity, and temporally evolving inflammatory signals [4, 13, 24].

Our results show that the culture undergoes progressive changes over time. During the first week in vitro, neurons identified by ChAT and βIII-tubulin immunoreactivity are readily observed together with GFAP-positive astrocytes and Iba1-positive microglia [27]. This mixed cellular composition provides a useful context for studying early glial responses in the presence of neuronal and non-neuronal cells. However, as expected for dissociated serum-containing primary cultures, neuronal profiles became less prominent over time, whereas non-neuronal cells progressively dominated the preparation. For this reason, the culture should not be viewed as biologically equivalent across all time points. Rather, the early phase is best interpreted as a mixed spinal cord culture, whereas later stages more closely represent a glia-dominant in vitro environment.

An important feature of this protocol is the preservation of astrocytic morphological heterogeneity, which is often reduced in purified or simplified monocultures. The coexistence of process-bearing, stellate, flattened, and pancake-like astrocytic forms is consistent with reports that neonatal rat spinal cord cultures contain multiple morphologically distinct astrocyte populations ([10, 33, 19]). These forms likely reflect astrocyte plasticity and underlying phenotypic diversity rather than a fixed classification, since process-bearing precursors can transition over time toward broader pancake-like phenotypes and culture conditions can influence their relative abundance ([19]). Although previous studies suggest that astrocyte classes may differ in lineage properties and membrane-associated phenotypes [10, 33], morphology alone is not sufficient to demonstrate functional specialization in the present platform. Thus, the main value of this model lies in preserving astrocytic heterogeneity within a mixed spinal cord environment, while the specific functional differences among these forms remain to be determined experimentally.

The inflammatory relevance of the model was examined using lipopolysaccharide, a well-established activator of TLR4 signaling [9, 23]. In spinal cord cultures, LPS has been used to activate glial inflammatory pathways and to promote the release of mediators associated with injury-related neuroinflammation [16, 31]. In the present study, exposure to 5 µg/mL LPS for 12 h increased nitrite levels in the culture supernatant, indicating activation of an inflammatory response. In this context, nitric oxide was considered a relevant readout because it reflects activation of iNOS-related inflammatory pathways downstream of glial stimulation [8]. Nevertheless, this finding should be interpreted within the limits of the model, since nitric oxide alone is not sufficient to demonstrate faithful reproduction of the secondary injury environment after SCI. In vivo, secondary injury is a complex and progressive process involving multiple cell types, endogenous danger signals, vascular dysfunction, excitotoxicity, oxidative imbalance, and temporal evolution of the inflammatory response [25, 41, 42]. Therefore, the present model is best interpreted as a controlled in vitro representation of a TLR4-mediated inflammatory component of secondary injury rather than as a complete reproduction of the post-traumatic spinal cord milieu.

The microglial response to LPS further supports the inflammatory competence of the model. We observed an increased number of amoeboid-like cells by phase-contrast microscopy, together with increased Iba1-positive and CD68-positive cell labeling after LPS exposure. These findings are consistent with a shift toward a more reactive inflammatory state and agree with previous studies showing that LPS promotes a less ramified and more amoeboid microglial morphology [8, 27, 36]. However, these results should be interpreted cautiously [ 37]. Primary spinal cord cultures are not fully quiescent systems, since dissociation, serum exposure, and adhesion to plastic can already induce a baseline reactive state in vitro [3, 31]. Thus, LPS likely enhanced a pre-existing culture-associated reactive profile rather than inducing activation de novo. In addition, CD68 in this context should be interpreted primarily as a marker of phagocytic or lysosomal activation rather than as a lineage marker alone. Accordingly, the observed increase in CD68-positive cells is most appropriately understood as evidence of enhanced phagocytic-like activation within the culture after inflammatory stimulation.

In addition to these marker changes, LPS treatment was associated with disruption of the organized pattern of cell bundle growth observed in control cultures. This suggests that inflammatory stimulation alters not only individual cell morphology but also the broader structural organization of the preparation. Because astrocytes play a central role in shaping the extracellular environment, these changes are likely related to glial remodeling within the mixed culture. In this context, the use of spinal cord-derived preparation is important, since previous work has shown that spinal and cortical astrocytes differ in marker expression and inflammatory behavior, emphasizing the need for tissue-specific models when evaluating glial responses and potential therapeutic interventions [7, 20].

The temporal profile of this culture provides distinct experimental windows rather than a single fixed biological state. During the early period, particularly up to DIV7, the preparation retains a mixed population of neurons and glial cells and is therefore suitable for studying early glial reactivity and testing acute anti-inflammatory interventions in a primary spinal cord context [2, 34]. At later time points, however, the culture progressively shifts toward a glia-dominant state, as expected for long-term serum-containing primary spinal cord cultures [19, 29]. Thus, maintenance of the culture up to 90 days should be interpreted as an opportunity to examine persistent glial survival, reactivity, and long-term phenotypic change in vitro, rather than as a full reproduction of the glial scar or the chronic secondary injury environment after SCI [4, 24, 38]. This interpretation preserves the value of the model while avoiding overstatement of its in vivo relevance.

Some limitations should be acknowledged. The population not labeled by the neuronal and glial markers used in this study remained unresolved and may include additional non-neuronal cell types commonly present in primary spinal cord preparations. In addition, long-term persistence of the culture does not imply preservation of full in vivo-like cellular identity across time. Finally, the present study did not directly test functional differences among astrocytic morphologies or cell-type-specific survival after inflammatory stimulation. These issues should be addressed in future work using expanded marker panels and functional essays.

In conclusion, this study presents a low-cost primary spinal cord culture that is compatible with the 3Rs principles and may be useful for screening anti-inflammatory compounds in a mixed spinal cord environment [43]. The characterization of cellular composition, astrocytic heterogeneity, and LPS-responsive inflammatory changes supports the use of this preparation as a practical in vitro platform for studying glial-associated TLR4-mediated responses. Although it does not reproduce the full systemic and pathological complexity of SCI, it provides an accessible system for investigating selected inflammatory mechanisms relevant to secondary injury and for supporting future neuroprotective studies.

## Data Availability

No datasets were generated or analysed during the current study.
