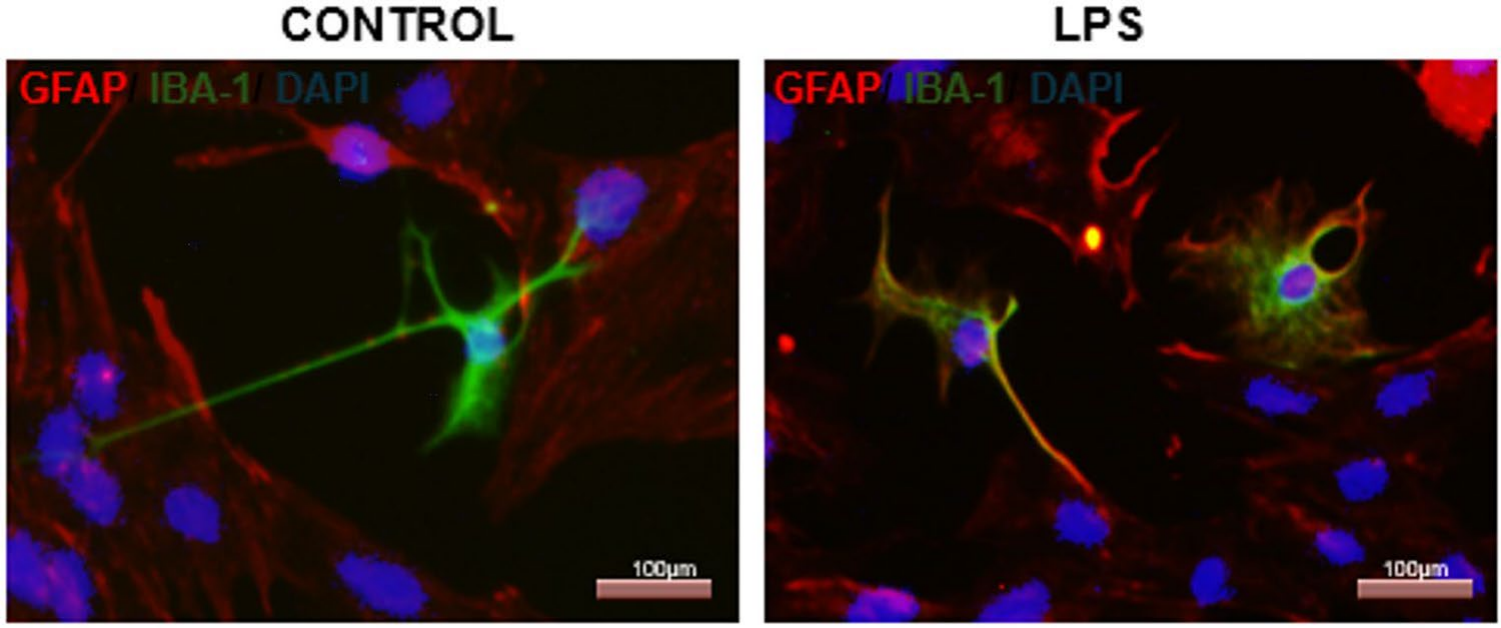# Correction: A Primary Spinal Cord Mixed Culture Method for In Vitro Analysis of Glial Heterogeneity and Inflammatory Responses

**DOI:** 10.1007/s11064-026-04805-8

**Published:** 2026-06-13

**Authors:** Cinthia Cristina de Oliveira Santos Costa, Catarina de Jesus Nunes, Ana Clara Neves Reis Pedreira, Silvia Lima Costa, Ravena Pereira do Nascimento

**Affiliations:** Laboratory of Neurochemistry and Cellular Biology, Institute of Health Sciences, Federal University of Bahia, Av. Reitor Miguel Calmon S/N, Salvador, 40231-300 BA Brazil

**Correction to: Neurochemical Research (2026) 51:167**

10.1007/s11064-026-04777-9

In this article, Figs. 7.0 and 7.1 appeared incorrectly and have now been corrected in the original publication. For completeness and transparency, the old incorrect versions are displayed below.

The original article has been corrected.

Incorrect Fig. 7


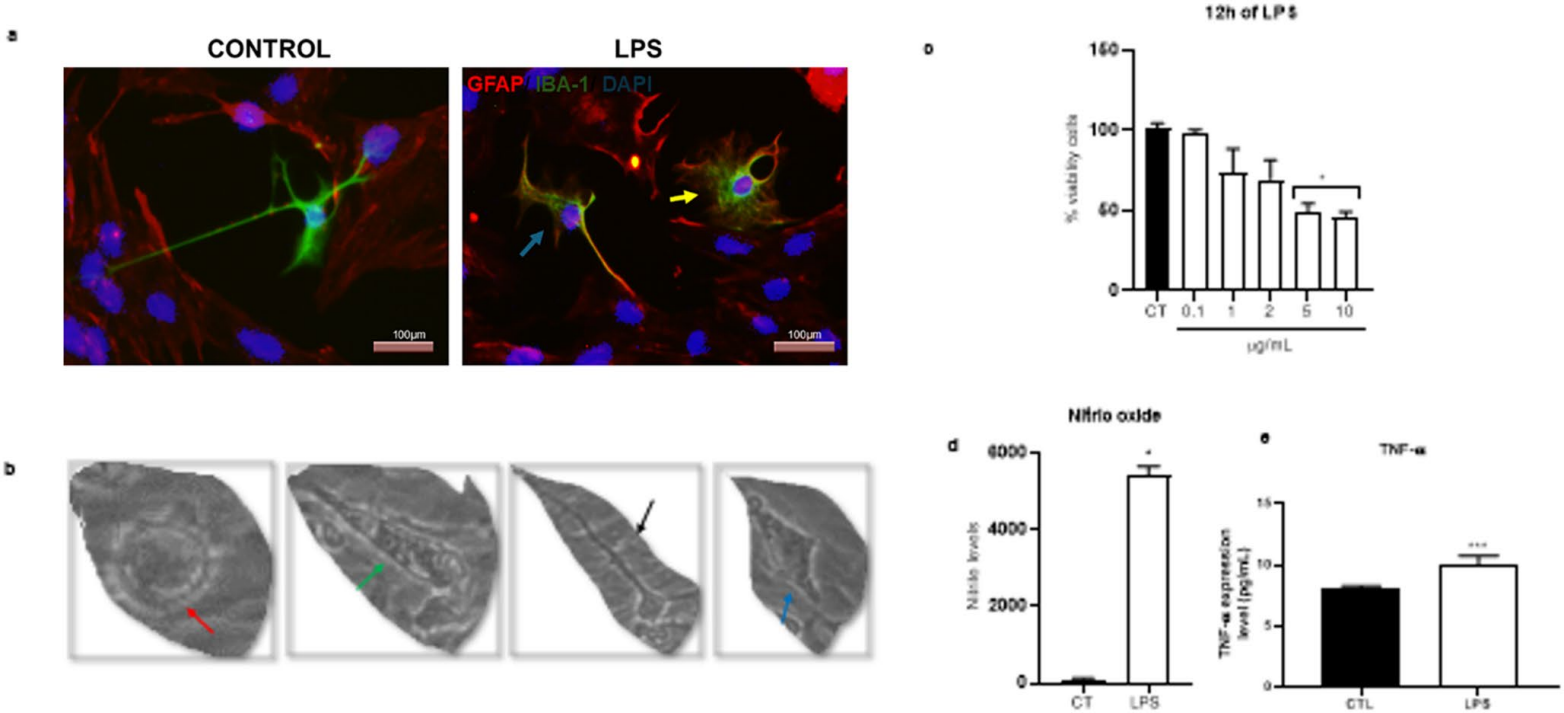


Incorrect Fig. 7.1